# Green Synthesis of Silver Nanoparticles Using *Olea europaea* Leaf Extract for Their Enhanced Antibacterial, Antioxidant, Cytotoxic and Biocompatibility Applications

**DOI:** 10.3390/ijms222212562

**Published:** 2021-11-22

**Authors:** Hanen Sellami, Shakeel Ahmad Khan, Ishaq Ahmad, Abdullah A. Alarfaj, Abdurahman H. Hirad, Ahmed E. Al-Sabri

**Affiliations:** 1 Laboratory of Treatment and Valorization of Water Rejects, Water Research and Technologies Center (CERTE), Borj-Cedria Technopark, University of Carthage, Soliman 8020, Tunisia; sellami_hanen@yahoo.fr; 2 Center of Super-Diamond and Advanced Films (COSDAF), Department of Chemistry, City University of Hong Kong, 83 Tat Chee Avenue, Kowloon, Hong Kong 999077, China; 3 Department of Physics, The University of Hong Kong, Hong Kong 999077, China; ahmadrai@hku.hk; 4 Department of Botany and Microbiology, College of Science, King Saud University, P.O. Box 2455, Riyadh 11451, Saudi Arabia; aalarfajj@ksu.edu.sa (A.A.A.); ahirad@ksu.edu.sa (A.H.H.); aalsabri@ksu.edu.sa (A.E.A.-S.)

**Keywords:** Ag NPs, *Olea europaea*, green synthesis, antioxidant, antimicrobial, cytotoxic

## Abstract

Herein, we report the green synthesis of silver nanoparticles (OE-Ag NPs) by ecofriendly green processes using biological molecules of *Olea europaea* leaf extract. Green synthesized OE-Ag NPs were successfully characterized using different spectroscopic techniques. Antibacterial activity of OE-Ag NPs was assessed against four different bacteriological strains using the dilution serial method. The cytotoxic potential was determined against MCF-7 carcinoma cells using MTT assay in terms of cell viability percentage. Antioxidant properties were evaluated in terms of 2,2-diphenyl-1-picrylhydrazyl (DPPH) free radical scavenging. Biocompatibility was further examined by incubating the synthesized NPs with hMSC cells for 24 h. The results were demonstrated that synthesized OE-Ag NPs presented excellent log_10_ reduction in the growth of all the tested bacterial strains, which as statistically equivalent (*p* > 0.05) to the standard antibiotic drug. Moreover, they also demonstrated excellent cytotoxic efficacy against the MCF-7 carcinoma cells compared to plant lead extract and Com-Ag NPs. Green synthesized OE-Ag NPs appeared more biocompatible to hMSC and 293T cells compared to Com-Ag NPs. Excellent biological results of the OE-Ag NPs might be attributed to the synergetic effect of NPs’ properties and the adsorbed secondary metabolites of plant leaf extract. Hence, this study suggests that synthesized OE-Ag NPs can be a potential contender for their various biological and nutraceutical applications. Moreover, this study will open a new avenue to produce biocompatible nanoparticles with additional biological functionalities from the plants.

## 1. Introduction

The emergence of drug resistance in different bacteria has become a significant public health problem. Currently, antibiotics have been used as an effective treatment against bacterial exposure owing to their satisfactory outcomes. However, their great use without maintaining prescription policies leads to increased antibiotic resistance in bacteria. Hence, multi-drug resistant (MDR) bacteria were defined as bacteria that acquired resistance to at least one antibiotic from three or more classes of antibiotics and are frequently responsible for infection associated with healthcare settings [1]. Moreover, an infection caused by MDR bacteria has become significantly challenging, especially in developing countries, and has been associated with high morbidity and mortality rates [2]. In addition, it has been estimated that infections by resistant bacteria may rise to 10 million death/year by 2050 more than any other causes and like the most global issues [3]. Therefore, the challenge of antibiotic resistance is becoming alarming, and the development of new alternatives antibacterial agents to combat has become an urgent need. Recently, nanotechnology has provided many multi-disciplinary research concepts in the scientific area, which can overcome this challenging situation [4]. This technology has led to the design and fabrication of nanomaterials and nanoparticles with exceptionally high surface area to volume ratio and small size, which determines their physical, chemical, mechanical, optical, electrical, solubility, and stability. Generally, several physical and chemical methods have been intensively used for synthesizing nanoparticles. However, these methods generally involve using a toxic solvent that would pollute the environment and strictly limit the use of NPs in scientific and technical fields. Hence, it is imperative to develop economic alternative processes for nanomaterials synthesizing for future biological applications [5]. Notably, in recent years, nanobiotechnology has been established as a new branch of nanotechnology. This technology combines biological systems with physical and chemical procedures to produce nano-sized particles with specific functions [6]. Hence, biological synthesis or ‘green’ synthesis of NPs has attracted significant attention due to their cost-effective, environmentally friendly, and resulting biocompatible nanoparticles [7]. Many naturally biological sources, including fungi, bacteria, plants, and algae, have been employed in the biosynthesis of nanoparticles. Although compared to other naturally available biological sources, plants have attracted much attention, and they are more suitable for the biosynthesis of NPs owing to their high stability, easily scalable, readily available, and no particular storage condition required. In typical NPs biosynthesis, plant extracts were used as reducing and stabilizing agents.

Moreover, these extracts are mainly composed of numerous secondary metabolites, such as sugars, polyphenols, flavonoids, and terpenoids. Their oxidation induces the reduction of metal ions and the formation of metallic NPs. Among the different metallic NPs, silver nanoparticles (Ag NPs) have been extensively explored for their exclusive properties (e.g., size and shape depending optical, electrical, and magnetic properties) [8,9,10]. On the other hand, Ag NPs have been shown to exhibit chemical stability, good conductivity, antibacterial and catalytic activities, as well as a cytotoxic effect on cancer cells [11]. In this regard, phytochemicals and secondary metabolites present in plant extracts can cap and reduce Ag^+^ ions to Ag^0^ [12]. Therefore, it is suitable for the large-scale synthesis of Ag NPs in a non-aseptic environment.

In this work, we have synthesized Ag NPs using biological molecules of leaf extract of *Olea europaea* L. *Olea europaea* L. belongs to the *Oleaceae* family, is one of the essential fruit trees widely cultivated in the Mediterranean region (approximately 98% of the world crop) [13]. Olive leaves are agricultural by-products that have attracted growing interest in the scientific and industrial community due to their health benefits and nutritional effects [14]. Hence, *Olea europaea* leaves were considered promising sources of bioactive compounds, mainly phenolic compounds. The major abundant compounds are Oleuropein and Hydroxytyrosol and other flavonoids, such as Apigenin-7-glucoside, Luteolin-7-glucoside, and Verbascoside [15,16]. The isolated bioactive compounds have been found to have antiviral [17], antimicrobial [18], antioxidant [19], anti-inflammatory [20], anticancer [16], antihypertensive [21] and hypoglycemic properties [22]. Thus, in the present research, Ag NPs were synthesized using *Olea europaea* phytomolecules as a reducing and stabilizing agent. The green synthesized OE-Ag NPs were further evaluated for their different applications, i.e., antioxidant, antibacterial, and anticancer.

## 2. Results and Discussion

### 2.1. Characterization

**Figure 1:**
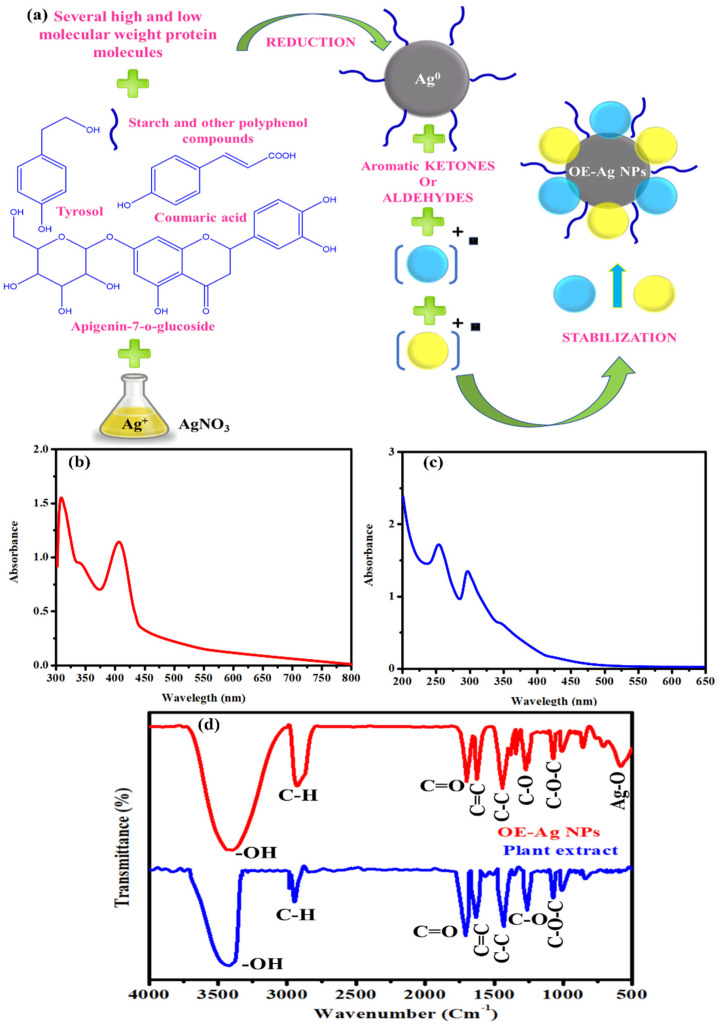
(**a**) Probable mechanism for the synthesis of OE-Ag NPs. (**b**,**c**) UV-Visible spectra of OE-Ag NPs and plant extract respectively. (**d**) FTIR analysis of plant extract and OE-Ag NPs.

*Olea europaea* is a rich source of different biological important secondary metabolites that can be used for the synthesis of biogenic nanoparticles as reducing and capping agents [23,24]. Therefore, we have employed the leaf extract of this plant as reducing and capping agents for the synthesis of biogenic silver nanoparticles. Figure 1a presents the possible mechanism for the synthesis of OE-Ag NPs using the plant leaf extract. Polyphenols and flavonoids present in the leaf extract might be involved in reducing the silver ions to metallic form following the redox reaction. Simultaneously, other secondary metabolites such as alkaloids, terpenoids, saponins present in the leaf extract might readily be stabilized the formed Ag^0^ by capping and finally converted into silver nanoparticles (OE-Ag NPs). Similar synthesis mechanism was also reported by Hussain et al. [25].

The chemical reaction between the dissolved silver ions and plant leaf extract was examined with a UV-Visible spectrophotometer. UV-Visible analysis revealed that upon reaction completion and formation of OE-Ag NPs, an absorption peak at 410 nm was observed due to the surface plasmon resonance phenomenon (Figure 1b) [26]. An additional absorption peak at 310 nm was also observed, which might be due to the adsorbed polyphenols. Literature shows that polyphenols usually produce UV-Visible absorption peaks in the ultra-violet region due to the presence of hydroxyl moieties [27,28,29,30]. On the other hand, plant leaf extract shows two UV-Visible absorption peaks (255 nm and 290 nm) in the ultraviolet region (Figure 1c). A slight blue shift in UV-Visible absorption peaks of plant leaf extract was observed compared to the reported by [31]. This might be attributed to the different phytomolecules present in the leaf extract which depends on the nature of soil and environments in which plant is grow.

FTIR analysis of both plant leaf extract and OE-Ag NPs was carried out further, and their spectra are shown in Figure 1d. FTIR spectra of plant leaf extract showing different IR peaks at 3400 cm^−1^ (-OH), 2920–2880 cm^−1^ (C-H), 1706 cm^−1^ (C=O), 1630 cm^−1^ (aromatic C=C), and several others [31] which indicates the presence of several phytomolecules. On the other hand, OE-Ag NPs are also presented a similar pattern of FTIR peaks with slight shifts in wavenumbers and intensity. Hence, FTIR results corroborated that green synthesized OE-Ag NPs are successfully capped and stabilized with the plant leaf extract’s secondary metabolites.

**Figure 2:**
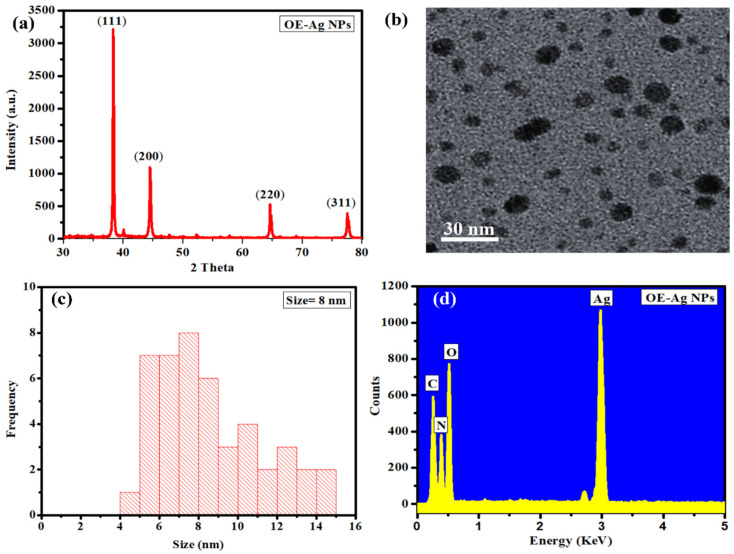
(**a**) XRD, (**b**) TEM, (**c**) Size distribution, and (**d**) EDX analysis of green synthesized OE-Ag NPs.

Further, the green synthesized OE-Ag NPs were characterized with XRD. XRD diffraction pattern indicates the sharp, narrow, and intense peaks corroborating the highly crystalline nature of the synthesized NPs (Figure 2a). Four diffraction peaks at 38.60^⁰^, 44.56^⁰^, 64.65^⁰^, and 77.65^⁰^ were observed, which correspond to crystal planes (111), (200), (220), and (311) respectively [32]. Figure 2b shows the TEM image of the green synthesized OE-Ag NPs. TEM analysis displays that the synthesized NPs are spherical in morphology with uniform distribution. TEM analysis shows that the average size of NPs is 8 nm (5 to 14 nm particle size range) (Figure 2c). Figure 2d shows the compositional analysis results carried out with EDX. EDX confirms that the synthesized NPs are mainly composed of silver. Besides, the spectra also show different EDX peaks associated with oxygen, nitrogen, and carbon. These peaks might be attributed to the surface adsorbed phytomolecules (alkaloids, polyphenols, flavonoids, etc.) from the plant leaf extract during capping. A consistency was observed in the results of FTIR and EDX analysis. Hence all characterization results confirm the successful fabrication of our desired nanoparticles.

### 2.2. Antibacterial Activity

**Figure 3:**
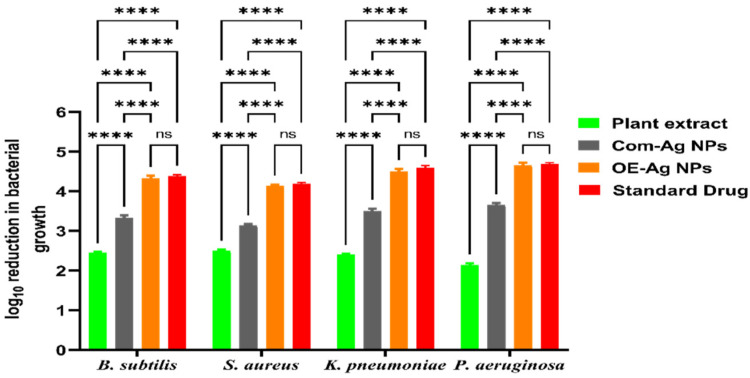
Antibacterial activity of green synthesized OE-Ag NPs in terms of log_10_ reduction in growth of all tested bacterial strains compared to plant leaves extract, Com-Ag NPs, and standard drug (**** and ns denote *p* < 0.0005, and *p* > 0.05, respectively).

Antibacterial activity of green synthesized OE-Ag NPs was assessed against Gram-positive and Gram-negative bacterial strains. The results demonstrated the highest antibacterial activity in terms of log_10_ reductions in bacterial growth with green synthesized OE-Ag NPs compared to a plant extract and Com-Ag NPs (Figure 3). Interestingly, the antibacterial activity presented by green synthesized OE-Ag NPs was comparable (*p* > 0.05) to the standard antibiotic drug. Moreover, plant leaves extract also displayed good antibacterial activity against all the microbial strains. The enhanced antibacterial activity of OE-Ag NPs might be due to the synergistic effect of NPs’s physical properties and adsorbed secondary metabolites on their surface from the plant extract, as evident from the results of FTIR and EDX analysis. A similar finding was also reported by [27,28,30,33,34,35,36].

**Figure 4:**
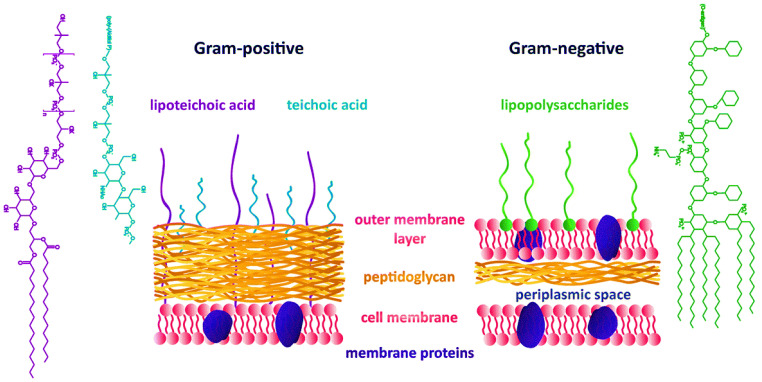
Comparison between the cell wall structure and composition of both Gram-positive and Gram-negative bacterial strains [39].

It has been observed that green synthesized OE-Ag NPs exhibited more efficiency in killing the Gram-negative bacterial strains than that of Gram-positive bacterial strains. This might be attributed to their easy penetration and more electrostatic attraction with surface bounds functional moieties (e.g., sulfur proteins) of the cell wall and cytoplasmic membrane of the Gram-negative bacterial strains compared to Gram-positive. The easy penetration of green synthesized OE-Ag NPs might have occurred because Gram-negative bacterial strains have thinner cell wall than Gram-positive (Figure 4) [37,38]. Moreover, both Gram-positive and Gram-negative bacteria differ in the structure and composition of their cell walls [39] as shown in Figure 4. The cell wall of Gram-positive bacteria comprises of multilayer channels of thick peptidoglycan, lipoteichoic acid and wall teichoic acid. Both lipoteichoic acid and wall teichoic acid are attached to peptidoglycan as well as cell membrane. On the other hand, Gram-negative bacteria cell wall consists of outer/inner membrane layers, thin layer of peptidoglycan, lipopolysaccharides, and gel like periplasm. The lipopolysaccharides are highly negatively charged macromolecules that are only present in outer membrane layers of Gram-negative bacterial strains [38,39].

#### Antibacterial Mechanism

**Figure 5:**
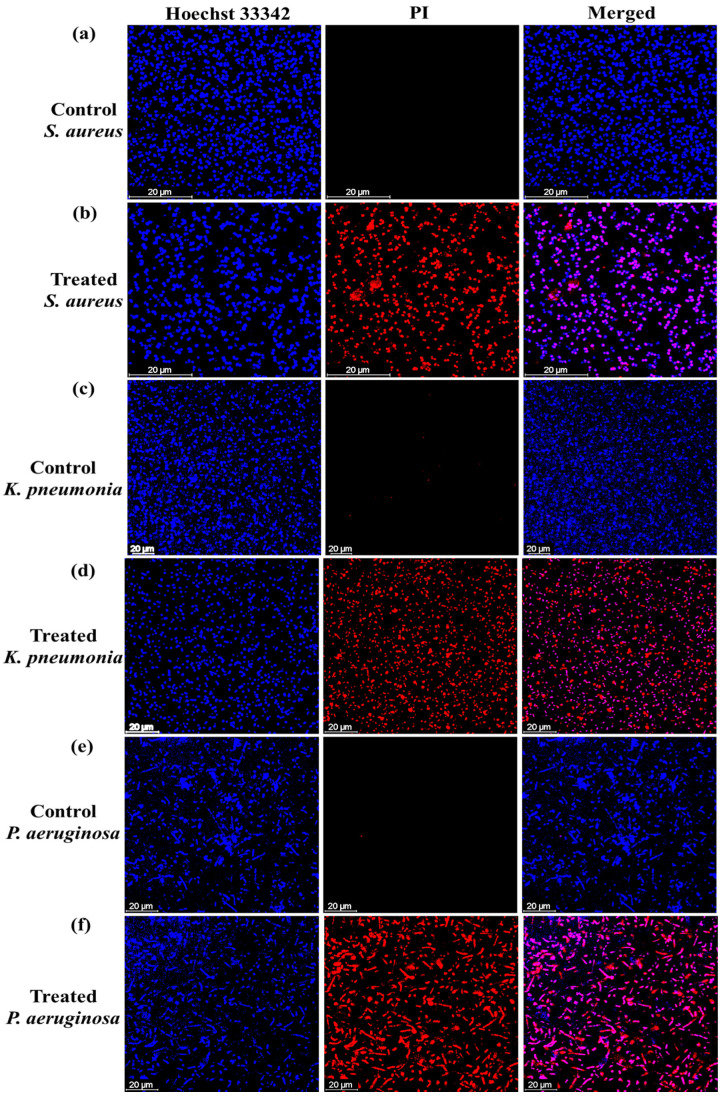
CLSM images of Live/dead bacteria. (**a**,**c**,**e**) control and (**b**,**d**,**f**) treated bacteria with green synthesized OE-Ag NPs.

The antibacterial mechanism of the green synthesized OE-Ag NPs was evaluated further with Live and dead staining assay. Live/dead stating assay was carried out against three bacteria *(S. aureus*, *K. pneumonia*, and *P. aeruginosa*) using a double staining kit (Hoechst 33,342 /PI). Both dyes are used for the differentiation of the live and dead bacteria cells. Hoechst 33,342 is a permanent membrane dye and can stain both live/dead cells upon intercalation with DNA [40]. On the other hand, PI is an impermeant membrane dye that can only penetrate bacteria cells when the cell membrane has been destroyed. Therefore, PI is used for the staining of dead cells. Figure 5 shows that untreated bacterial strains only stained with Hoechst 33,342, indicating that the bacteria cells live, and their cell membrane is intact. On the other hand, treated bacteria demonstrated red fluorescence displaying that their cell membrane has been destroyed by the action of green synthesized OE-Ag NPs. These results revealed that one possible reason behind the antibacterial action of green synthesized OE-Ag NPs is the disruption of bacteria cell membrane integrity.

**Figure 6:**
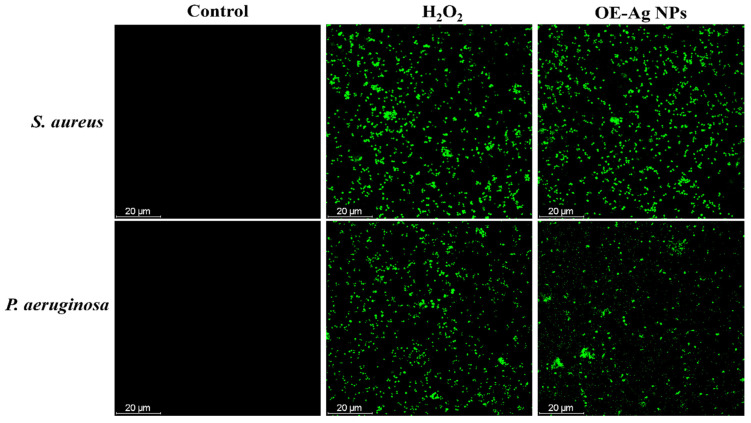
Intracellular ROS generation investigation by CLSM in bacteria (*S. aureus* and *P. aeruginosa*) treated with green synthesized OE-Ag NPs in comparison to H_2_O_2_.

After confirmation of bacterial cell deaths by physical damage of their cell membrane with the green synthesized OE-Ag NPs, we further hypothesized that bacterial cell death might also be induced by the oxidative stress produced by the reactive oxygen species (ROS) such as hydroxyl radicals OH^•^, superoxide ions O_2_^–•^, H_2_O_2_, and hydroperoxyl radicals [40]. It has been reported that Ag-based materials and nanoparticles have the ability to generate ROS upon interaction with microbial cells [41,42,43]. To get more insight into the antibacterial mechanism of the green synthesized OE-Ag NPs, we further performed an intracellular ROS measuring experiment using CellROX^®^Green staining kit. Figure 6 displayed that no intracellular ROS generation has been taken place in untreated bacteria. However, maximum ROS generation has been observed in all tested bacteria treated with H_2_O_2_. Similar ROS intracellular generation has also experimented with all bacteria treated with green synthesized OE-Ag NPs. These findings further corroborated that the bacteria cell death also resulted from oxidative stress produced by the green synthesized OE-Ag NPs.

After examining the rupture of cell membrane integrity and the generation of intracellular oxidative stress caused by ROS species, we can suggest a mechanism for Bacterial cell death caused by green produced OE-Ag NPs. As a consequence, the OE-Ag NPs exhibited superior antibacterial activity as a result of physical and oxidative degradation, which resulted in the production of various intracellular alterations. (1) The interaction of OE-Ag NPs with cell membrane proteins and other biomacromolecules such as lipopolysaccharides, etc., results in the breakdown of the cell membrane. (2) Disintegration of the cell membrane increased the membrane’s permeability for nanoparticle penetration. (3) Membrane permeabilization may also be triggered the leakage of intracellular function. (4) Following their penetration, the OE-Ag NPs may interact with various cellular organelles, causing oxidative stress and further impairing their physiological activities. All of these factors may contribute to the OE-Ag NPs’ ability to kill Gram-positive and Gram-negative bacteria.

### 2.3. Antioxidant Potential

**Figure 7:**
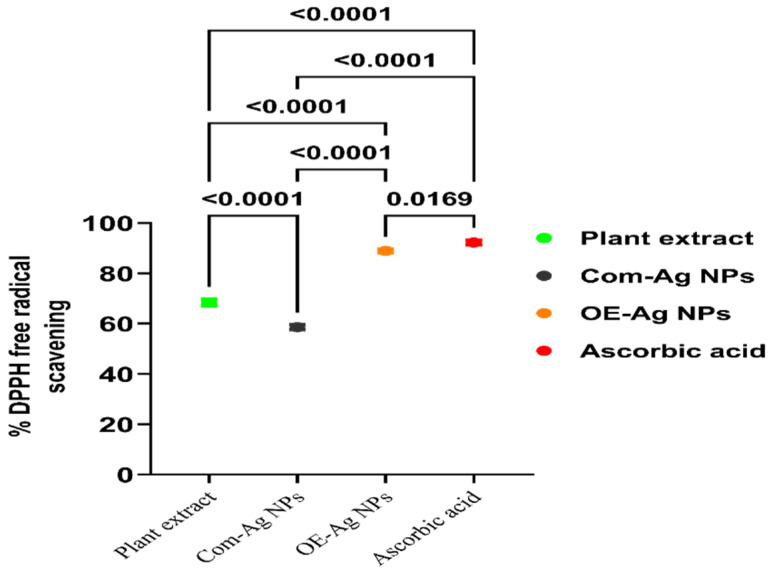
Antioxidant potential evolution in terms of percentage of DPPH free radical scavenging with green synthesized OE-Ag NPs compared to plant leaf extract, Com-Ag NPs, and standard antioxidant (ascorbic acid).

The green synthesized OE-Ag NPs’ antioxidant potential was evaluated in terms of DPPH free radical scavenging compared to plant leaves extract, Com-Ag NPs, and standard ascorbic acid. The results were displayed the maximum antioxidant activity with green synthesized OE-Ag NPs compared to plant extract and Com-Ag NPs, however comparable to standard ascorbic acid (Figure 7). On the other hand, plant leaf extract significantly scavenged the DPPH free radical and presented good antioxidant potential compared to Com-Ag NPs. The good antioxidant activity of the plant leaves extract might be attributed to the presence of electron-rich secondary metabolites such as phenolics, flavonoids, etc., [44,45,46,47]. Secondary metabolites such as phenolics and flavonoids are well-acknowledged to present antioxidant properties [48,49,50]. In the case of OE-Ag NPs, their enhanced DPPH scavenging ability compared to Com-Ag NPs might also be due to the presence of these secondary metabolites on their surface. Similar findings of enhanced antioxidant activity upon adsorption of these secondary metabolites on the surface of green synthesized NPs and upon metal chelation with phytomolecules compared to alone metal-ligand were also reported by [51,52,53,54,55].

### 2.4. Cytotoxic Propensity

**Figure 8:**
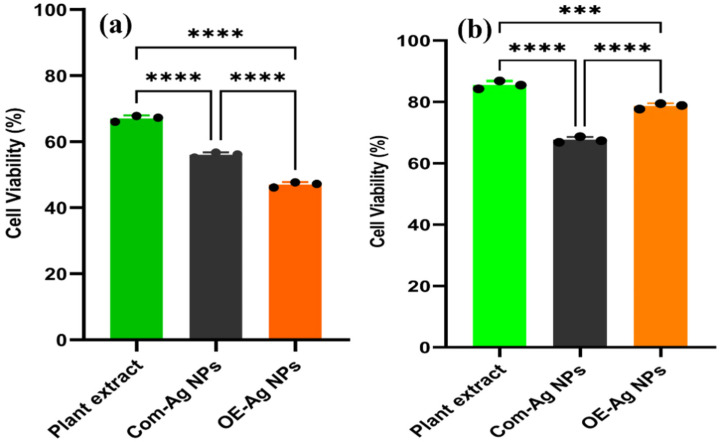
(**a**) Cytotoxic propensity against MCF-7 carcinoma cells and (**b**) biocompatibility against hMSC cells after incubation for 24 h with OE-Ag NPs, plant leaf extract and Com-Ag NPs (**** *p* < 0.0001, *** *p* < 0.0005).

The cytotoxicity propensity of the green synthesized OE-Ag NPs was determined in terms of cell viability percentage of MCF-7 carcinoma cell lines using MTT assay compared to plant extract and Com-Ag NPs. Findings exhibit that the superior cytotoxic effect was induced by the green synthesized OE-Ag NPs than both plant leaf extract and Com-Ag NPs (Figure 8a). On the other hand, Com-Ag NPs presented more cytotoxic efficacy against the MCF-7 carcinoma cells compared to plant leaf extract. It was worthy to note that plant leaf extract also demonstrated good killing efficacy against MCF-7 cells which can be attributed to the presence of cancer killing secondary metabolites (polyphenols, flavonoids, alkaloids, terpenoids, saponins, etc.) [56,57]. On the other hand, enhanced cytotoxic propensity of OE-Ag NPs compared to Com-Ag NPs might be due to the presence of biological active secondary metabolites of plant leaf extract on their surface. Mona et al. has also reported the similar enhanced cytotoxic efficacy of biogenic silver nanoparticles synthesized by lichens [26].

### 2.5. Biocompatibility Analysis

Biocompatibility of the nanoparticles is the utmost desire for their application in biological system. Therefore, we further evaluated synthesized OE-Ag NPs for their biocompatibility with normal cell line hMSC using MTT assay compared to plant leaf extract and Com-Ag NPs. The results are presented in Figure 8b. Results were demonstrated that plant leaf extract displayed excellent biocompatibility presenting superior cell viability percentage (85.51%). While green synthesized OE-Ag NPs exhibited good biocompatibility with hMSC cell lines and shown 78.66% cell viability percentage. On the other hand, least biocompatibility was shown by the Com-Ag NPs.

**Figure 9:**
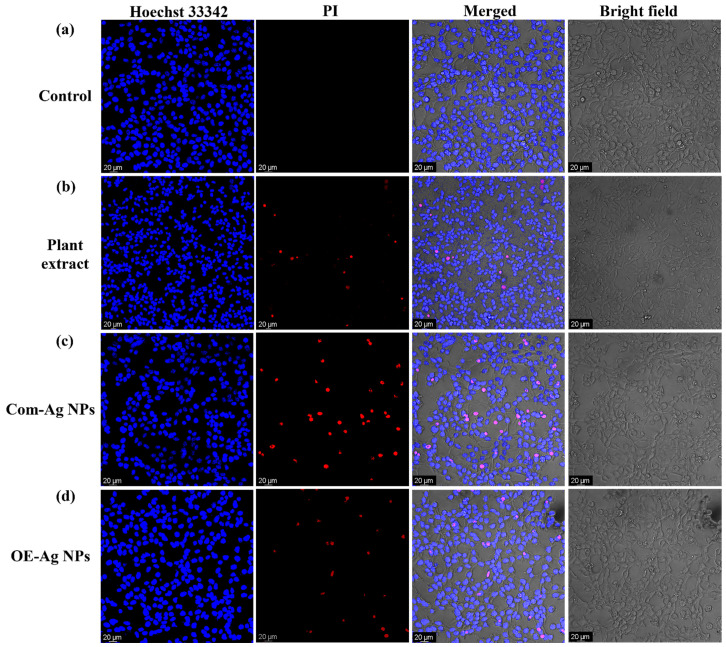
CLSM images of live/dead 293T cells. (**a**) Control (untreated) and treated with (**b**) plant extract, (**c**) Com-Ag NPs, and (**d**) OE-Ag NPs.

Using the live/dead staining approach, we further evaluated the cytobiocompatibility of the OE-Ag NPs with 293T cell line in comparison to the plant leaf extract Com-Ag NPS. Figure 9a–d illustrates the findings. The results revealed that plant leaf extract and OE-Ag NPs had the least detrimental impact on 293T cells, resulting in fewer cells dying. In comparison, chemically generated Com-Ag NPs were more lethal, and a significant number of cells seemed to be perished (Red). The enhanced cytobiocompatibility of green synthesized OE-Ag NPs with hMSC cells and 293T cells might be resulted because of the existence of biological active and biocompatible secondary metabolites of the plant leaf extract. The similar finding on the biocompatible nature of plant leaf extract and enhanced cytobiocompatibility of the nanoparticles after the inclusion of secondary metabolites on their surface were also reported by [58,59].

## 3. Materials and Methods

### 3.1. Chemicals and Collection of Plant Materials

Analytical grade chemicals and chemically synthesized silver nanoparticles (Com-Ag NPs) were purchased from Sigma-Aldrich. Experiments were carried out on leaves of the Chetoui cultivar of *Olea europaea* L., cultivated in Borj El Amri in the North of Tunisia. Fresh green leaves were collected until the end of olive morphology (February 2019). *Olea europaea* leaves were identified and authenticated by Professor Fathi Ben Amar at the olive institute of Sfax, Tunisia.

### 3.2. Plant Extract Preparation and OE-Ag NPs Synthesis

Fresh leaves of *Olea europaea* were washed with deionized water (DI) to remove dust particles, plankton, and insects, and then dried under shady at room temperature (25–30 °C). The dried leaves were milled into powder using a commercial grinder. The plant leaves powder was stored in glass bottles for future use. The preparation of plant extract and synthesis of OE-Ag NPs was carried out following the procedure reported by [30]. Ten g of the ground leaves powder of *Olea europaea* was added to 100 mL of DI water. Then, the aqueous mixture was heated to boil with rigorous stirring for 5 min. The boiled mixture was filtered with filter paper to get a yellowish-colored extract. Then, the leaves extract was used as a reducing and capping agent to convert silver salt to its metallic form. A total of 1 mM of AgNO_3_ was added to 25 mL of leaves extract of *Olea europaea*. Afterward, the mixture was heated at 80 °C for 65 min with constant stirring to obtain a dark brown dispersion of OE-Ag NPs. After centrifugation for 15 min at 15,000 rpm, the obtained OE-Ag NPs were washed with DI water three times and dried in an oven at 70 °C.

### 3.3. Characterization of OE-Ag NPs

The green synthesis OE-Ag NPs were characterized using different spectroscopic techniques. X-ray Diffraction (XRD) was performed using Bruker D_2_ PHASER with LYNXEYE XE-T detector diffractometer (Haidian, Beijing, China) operating at 40 kV and 30 mA with Cu Kα radiation (k =1.54056 A°) to test the crystallinity and purity of OE-Ag NPs. Energy-dispersive X-ray spectroscopy using Thermo Fisher Scientific Ultradry (Madison, WI, USA) was performed to determine the elemental composition of the green-synthesized OE-Ag NPs. A transmission electron microscope (TEM) (Jeol, 5910 LV, Tokyo, Japan) was carried out to determine the morphology of the synthesized Ag NPs. Fourier Transform Infra-Red spectra (FTIR) (Bruker, Germany. Model: Vertex 70) analysis was performed to determine whether phytomolecules of plant leaves extract were involved or not in the green synthesis of OE-Ag NPs. UV–visible analysis was carried out using a spectrophotometer (Shimadzu 1700, Columbia, MD, USA).

### 3.4. Antioxidant Activity

Antioxidant activity of the green synthesis OE-Ag NPs was determined by using a DPPH free radical scavenging assay [5]. In a typical experiment, DPPH solution (0.1 mM) in ethanol was mixed with different concentrations (500 µg/mL) of each sample (OE-Ag NPs, plant extract, Com-Ag NPs, and standard) that were prepared separately. Then, the reaction mixtures were stirred for 10 min and kept in the dark at room temperature for 1 h. After incubation, the absorbance was measured with a UV-Visible spectrophotometer at 517 nm. The percentage scavenging activity was calculated using the following formula:

% DPPH free radical scavenging = [(A_b_ − A_s_)/A_b_] × 100, (1)

where A_s_ is the absorbance of the sample and A_b_ is the blank absorbance (only DPPH solution).

### 3.5. Antibacterial Activity

The synthesized OE-Ag NPs were scrutinized for their antibacterial activity against two Gram-negative bacterial strains [*Pseudomonas aeruginosa* (ATCC^®^27853TM) and *Klebsiella pneumonia* (ATCC^®^ 13,883)] and two Gram-positive bacterial strains [*Staphylococcus aureus* (ATCC^®^ 15,564) and *Bacillus subtilis* (ATCC^®^ 6051)] using the serial dilution method as described by [60]. In general, the strains of bacteria (5 × 10^5^ CFU/mL) were seeded onto separate blood agar plates and then cultured for 24 h at 37 °C. After several bacterium colonies were grown on the plates, they were diluted with phosphate buffer saline (PBS). Their cell density was maintained to 1 × 10^7^ colony forming units (CFU) per mL. Following that, 10 µL of each bacterial culture was separately added to the wells of a 24-well microtiter plate with 1.0 mL of Mueller–Hinton broth (MHB). For each well, the final concentration of each bacterium was 1 × 10^5^ CFU/mL. A 50 µL of each sample solution at 250 µg/mL concentration was then transferred to separate wells and incubated at 37 °C for 24 h. The bacterial species were then counted in the wells using the serial dilution plate counting method. The antibacterial propensity was expressed in the form of log_10_ reduction in bacterial growth using the following formula:

Log_10_ reduction = log_10_ (CFU_BI_) − log_10_ (CFU_AI_), (2)

where CFU_BI_ and CFU_AI_ are the CFU of bacterial strains before and after 24 h of incubation, respectively, with the treatment of sample solutions. Standard antibiotic drug used in this study was Cefradine.

### 3.6. Live/Dead Bacterial Staining Assay

Live and dead bacterial staining assay was carried out using a confocal laser scanning microscope (CLSM, FV-1200, Olympus, Tokyo, Japan) to confirm the antibacterial activity of green synthesized OE-Ag NPs. The assay was performed following the methods reported by Choi et al. [61]. Briefly, two nucleic dyes, Hoechst 33,342 (membrane-permeant) and propidium iodide (PI; membrane-impermeant), were used for staining the live (green) and dead (red) bacteria, respectively. Each bacterium was cultured in nutrient broth in an orbital shaker for 24 h at 37 °C to reach the stationary phase, which was consists of approximately 10^5^–10^6^ colony forming units (CFU) per ml. After incubation, each bacteria strain was inoculated into sterilized cover glass coated with poly-L-lysine in a 24-well plate and then incubated for 1 h for bacterial cells attached to the cover glass. The suspended bacterial cells were then discarded, and each cover glass was gently rinsed three times with a saline solution. For the treatment, each bacterium cells on the cover glass were incubated with green synthesized OE-Ag NPs (250 μg/mL) and then incubated for 24 h at 37 °C. Bacteria cells on cover glass were then stained with an alive and dead bacterial viability kit in accordance with the manufacturer’s recommendations. Dead and live bacterial cells were analyzed with CLSM using an excitation wavelength of 493 nm and 350 nm for PI and Hoechst 33,342 and an emission wavelength of 636 nm and 461 nm for PI and Hoechst 33,342, respectively. We only considered green synthesized OE-Ag NPs for live/dead staining assay as they presented excellent antibacterial properties in terms of Log_10_ reductions.

### 3.7. Intracellular ROS Generation Investigations

The CellROX^®^Green staining was further used to examine the death of bacterial species caused by intracellular ROS production. In brief, bacterial species (*Staphylococcus aureus* and *Pseudomonas aeruginosa*) at 1 × 10^7^ CFU/mL with 70 μL of produced OE-Ag NPs at a concentration of 250 μg/mL and incubating at 37 °C for 24 h. Following that, the microbial cells were treated for an additional 30 min at 37 °C with CellROX^®^Green (5 μm). Following that, CLSM was utilized to acquire CLSM images at 485 nm absorption and 520 nm emission wavelengths. To assess microbial cells’ ability to generate reactive oxygen species (ROS), the results of cells treated with NPs were compared to those treated with 1 mm H_2_O_2_ (positive control) and untreated cells (negative control).

### 3.8. Anticancer Activity

The OE-Ag NPs were tested for their anticancer activity against MCF-7 (breast cancer cells) by MTT (3-(4, 5-dimethylthiazol-2-yl)-2, 5-diphenyltetrazolium bromide) colorimetric assay [30]. The MCF-7 carcinomatous cells were kept in Dulbecco’s Modified Eagle’s Medium (DMEM) in a humidified atmosphere comprising 5% CO_2_ and 95% air at 37 °C. To get the cell confluency (5 × 10^8^ cells/well), MCF-7 cells were cultured in a 96-microtiter plate containing 100 µL of DMEM for 24 h at 37 °C in 5% CO_2_. Different concentrations (1, 5, 10, 15, 30, 60, and 120 µg/mL) of green synthesized OE-Ag NPs were prepared in cell culture media and then mixed with cancer cells, and the plate was further incubated for 24 h at 37 °C. Cells without OE-Ag NPs were served as a negative control, and cells with the standard drug (doxorubicin) were served as a positive control. Afterward, the plate was centrifuged to remove the supernatant and subsequently washed with phosphate buffer saline solution. A total of 15 µL of MTT reagent at the concentration of 0.5 mg/mL was added to each well, and the plate was then incubated for 4 h at 37 °C. After incubation, 150 µL of DMSO were added to each well to solubilize the formazan crystals and kept on a shaker for 10 to 15 min. The absorption of the formazan product was determined using a spectrophotometer at 570 nm. The percentage of cell viability was calculated using the following formula:

Cell viability = [OD _sample_/OD _control_] × 100, (3)

### 3.9. Biocompatibility Analysis

The similar experiment was repeated as stated in Section 3.8. The only difference was cell lines. In this study, we used hMSC line.

Then, using the fluorescent staining approach, we employed a live/dead double staining kit to determine the viability of 293T cell line. The procedure was repeated until cancer cells were treated with different samples (10 µL at a concentration of 120 µg/mL) and incubated after treatment. After that, each well was incubated with the staining solution (4 g/mL) for 20 min at 37 °C. The images were taken using confocal laser scanning microscopy (CLSM) (535/617 nm and 361/497 nm excitation/emission wavelengths for PI and Hoechst 33,342, respectively).

### 3.10. Statistical Analysis

All biological assays were performed at three replicates, and results are presented in Mean ± SD. The significance level at *p* < 0.05 between the results was determined by applying One-way and two-way ANOVA.

## 4. Conclusions

Herein, we successfully reported the green synthesis of silver nanoparticles (OE-Ag NPs) by ecofriendly green processes using biological molecules of *Olea europaea* leaf extract. Green synthesized OE-Ag NPs were successfully characterized using different spectroscopic techniques. The results were demonstrated that synthesized OE-Ag NPs presented excellent log_10_ reduction in the growth of all the tested bacterial strains, which as statistically equivalent (*p* > 0.05) to the standard antibiotic drug. Moreover, they also demonstrated excellent cytotoxic efficacy against the MCF-7 carcinoma cells compared to plant lead extract and Com-Ag NPs. Green synthesized OE-Ag NPs appeared more biocompatible to 293 T and hMSC cells compared to Com-Ag NPs. Excellent biological results of the OE-Ag NPs might be attributed to the synergetic effect of NPs’ properties and the adsorbed secondary metabolites of plant leaf extract. Hence, this study suggests that synthesized OE-Ag NPs can be a potential contender for their various biological and nutraceutical applications. More study must be carried out to determine the dose-dependent biocompatibility in vitro as well as in vivo. Moreover, this study will open a new avenue to produce biocompatible nanoparticles with additional biological functionalities from the plants.

## Data Availability

The data are not publicly available.
